# Astaxanthin Sensitizes Low SOD2-Expressing GBM Cell Lines to TRAIL Treatment via Pathway Involving Mitochondrial Membrane Depolarization

**DOI:** 10.3390/antiox11020375

**Published:** 2022-02-13

**Authors:** Juhyun Shin, Arti Nile, Ramesh Kumar Saini, Jae-Wook Oh

**Affiliations:** 1 Department of Stem Cell and Regenerative Biotechnology, Konkuk University, Seoul 05029, Korea; junejhs@konkuk.ac.kr (J.S.); aartibmahajan@gmail.com (A.N.); 2 Department of Crop Science, Konkuk University, Seoul 05029, Korea; saini1997@konkuk.ac.kr

**Keywords:** astaxanthin, glioblastoma multiforme, reactive oxygen species, mitochondrial potential, apoptosis

## Abstract

Carotenoids have been suggested to have either anti- or pro-oxidative effects in several cancer cells, and those effects can trigger an unbalanced reactive oxygen species (ROS) production resulting in an apoptotic response. Our study aimed to evaluate the effect of the well-known carotenoid 3, 3′-dihydroxy-β, β’-carotene-4, 4-dione (astaxanthin, AXT) on glioblastoma multiforme (GBM) cells, especially as a pretreatment of tumor necrosis factor (TNF)-related apoptosis-inducing ligand (TRAIL), that was previously shown to increase ROS and to induce apoptosis in cancer cells. We found that AXT by itself did not trigger apoptosis in four investigated GBM cell lines upon a 24 h treatment at various concentrations from 2.5 to 50 µM. However, in U251-MG and T98-MG GBM cells, pretreatment of 2.5 to 10 µM AXT sensitized cells to TRAIL treatment in a statistically significant manner (*p* < 0.05) while it did not affect CRT-MG and U87-MG GBM cells. We further compared AXT-sensitive U251-MG and -insensitive CRT-MG response to AXT and showed that 5 µM AXT treatment had a beneficial effect on both cell lines, as it enhanced mitochondrial potential and TRAIL treatment had the opposite effect, as it decreased mitochondrial potential. Interestingly, in U251-MG, 5 µM AXT pretreatment to TRAIL-treated cells mitochondrial potential further decreased compared to TRAIL alone cells. In addition, while 25 and 50 ng/mL TRAIL treatment increased ROS for both cell lines, pretreatment of 5 µM AXT induced a significant ROS decrease in CRT-MG (*p* < 0.05) while less effective in U251-MG. We found that in U251-MG, superoxide dismutase (SOD) 2 expression and enzymatic activity were lower compared to CRT-MG and that overexpression of SOD2 in U251-MG abolished AXT sensitization to TRAIL treatment. Taken together, these results suggest that while AXT acts as an ROS scavenger in GBM cell lines, it also has some role in decreasing mitochondrial potential together with TRAIL in a pathway that can be inhibited by SOD2.

## 1. Introduction

Glioblastoma multiforme (GBM) is the most common and aggressive form of brain tumor. Characteristically known for their highly heterogeneous tumor features, even their origin is speculated to be heterogeneous, that is, to develop from mutated neural stem cells, glial progenitors, or astrocytes [1], contributing to the complexity involved in their prevention and treatment. While the occurrence of brain or other nervous system cancers is relatively low compared to other cancers (1.3% of all new cases of cancer in 2021, USA), the 5-year survival rate is as low as 32.6%, with no significant improvement since 1992 (NIH Cancer Statistics, https://seer.cancer.gov/statfacts/html/brain.html accessed on 13 December 2021). To date, while cutting-edge treatments involving immunotherapy and individualized gene editing are still under development, GBM treatments rely mainly on surgical procedures, radiation, and chemical therapies that involve the removal of brain tissue [2]. Therefore, GBM is not only difficult to treat, but its current treatments have devastating side effects, underlining the necessity to develop novel therapeutic treatments for GBM.

Tumor necrosis factor (TNF)-related apoptosis-inducing ligand (TRAIL) was first discovered by its homology to other TNF superfamily proteins [3]. Together with its receptors (TRAIL-Rs), it was originally proposed as a target to develop anti-cancer treatment, as it selectively triggers apoptosis in various cancer cells. While the core mechanism of TRAIL-induced apoptosis has been well defined early on [4], the factors that result in this differential TRAIL response was not well defined besides the straightforward presumption that this selectivity might be because TRAIL-Rs are usually more abundant in cancer cells compared to normal cells [5]. However, as reviewed recently, only a few drugs are in clinical trials, and the results are limited [6]. This is partially due to the fact that after its identification, it was found that several cancer cell lines and primary cancer cells gained TRAIL resistance [7]. Moreover, it has been reported that TRAIL-R2 can also have pro-oncogenic properties, such as in cancer cells with oncogenic mutations in the Kirsten rat sarcoma virus (KRAS) [8] or wild-type p53 gene [9]. Therefore, a better understanding of the cancer cell TRAIL/TRAIL-R apoptosis mechanism and gained resistance will be crucial for the development of TRAIL-related anti-cancer drugs [10].

Reactive oxygen species (ROS) are highly reactive chemicals occurring in eukaryotic cells through aerobic metabolism. ROS include peroxides, hydroxyl radicals, singlet oxygen, and alpha oxygen. While ROS increase is usually linked to cell damage in healthy cells, they are also known to act as signaling molecules in several cell pathways [11]. As cancer cells maintain a relatively high ROS environment promoting oncogenic proliferation, strategical ROS modulation (from ROS increase inducing apoptosis to ROS decrease to prevent proliferation) has been proposed for cancer treatment [12].

Carotenoids are pigments that are produced in various plants, algae, bacteria, and fungi and are known for their ability to control cellular ROS by having both antioxidant [13] and pro-oxidant properties [14]. Both properties are known to dysregulate cancer cell ROS, resulting in cancer cell-specific apoptosis while having protective ability in nearby healthy cells [15,16]. 3, 3-dihydroxy-β, β’-carotene-4, 4-dione (Astaxanthin, AXT) is a carotenoid that is known for its strong antioxidant properties [17]. Like all carotenoids, AXT is not biosynthesized in the human body, and its uptake commonly relies on food sources such as fishes or other marine organisms [18]. In 2019, the European Food Safety Authority’s (EFSA) Scientific Panel on Additives and Products or Substances used in Animal Feed (FEEDAP) derived an acceptable daily intake (ADI) of 0.2 mg astaxanthin/kg bw for human consumption [19]. AXT was previously reported to have anti-cancer properties in various cancer cell types, such as oral, bladder, colon, liver, lung, breast cancer, and leukemia. These properties involve various pathways resulting in inhibition of cell proliferation, angiogenesis, inflammation signaling, cell cycle progression, and/or triggering apoptosis [20,21]. More recently, it was shown to inhibit metastasis and promote apoptosis in colon cancer cell lines [22,23] and induce necroptosis in gastric cancer cell lines [24].

Several studies have shown that there is a cross-talk between the TRAIL-triggered apoptotic pathway and intracellular ROS in various cancer cell lines, including pancreatic cancer cell lines [25], cervical cancer cell line [26], melanoma cell line [27], and GBM cell line [28]. However, the role of AXT in GBM cells and its effect on TRAIL treatment have not been well studied.

Therefore, the aim of our study is to investigate the effects of AXT and TRAIL treatment on the viability of GBM cell lines.

## 2. Materials and Methods

### 2.1. GBM Cell Lines, Media, and Culture Conditions

Human U251-MG and U87-MG GBM cell lines were obtained from Dr. Benveniste EN (University of Alabama at Birmingham, Birmingham, AL, USA). T98G human astroglioma GBM cell line was purchased from the American Type Culture Collection (ATCC CRL-1690, Manassas, VA, USA) and CRT-MG GBM cells were established as previously described [29]. Cells were determined to be mycoplasma-free based on the Biomax Mycoplasma PCR Analysis Kit (Biomax Inc., Daejeon, Korea). U251-MG, U87-MG, and T89G-MG cell lines were grown in minimum essential medium (MEM; Welgene, Gyeongsan, Korea) supplemented with 1% of 100 mM sodium pyruvate (Welgene, Gyeongsan, Korea), 1% 100× penicillin/streptomycin (Welgene, Gyeongsan, Korea), and 10% fetal bovine serum (FBS; Welgene, Gyeongsan, Korea). CRT-MG cells were grown in Dulbecco’s modified Eagle’s medium (DMEM; Welgene, Gyeongsan, Korea) supplemented with 1% 100× penicillin/streptomycin and 10% FBS. Cells were cultured in 100 mm cell culture dishes in an incubator at 37 °C and 5% CO_2_ and sub-cultured every 3 days after they reached approximately 90% confluence. The cells were not cultured for more than 15 passages.

### 2.2. Cell Treatment

Astaxanthin (Merck, Darmstadt, Germany) was dissolved in dimethyl sulfoxide (DMSO; Sigma-Aldrich, St. Louis, MO, USA). Prior to treatment, the cells were counted using a hemocytometer and plated in cell culture plates. After overnight incubation, the plates were gently washed once with Dulbecco’s phosphate-buffered saline (DPBS; Welgene, Gyeongsan, Korea), and FBS-free media were added. AXT or DMSO were treated accordingly as a control, and plates were incubated at 37 °C. For AXT pretreatment and TRAIL treatment, cells were treated with AXT for 3 h at 37 °C prior to treatment with recombinant human sTRAIL/Apo2L protein (TRAIL; Peprotech, Rocky Hill, NJ, USA) dissolved in H_2_O.

### 2.3. WST-1 Cell Viability Assay

U251-MG, U87-MG, CRT-MG, and T98G-MG cells were plated in 96-well plates at a density of 5 × 10^3^ cells/well. After 24 h treatment with AXT, TRAIL, and 0.1% DMSO as a control, a cell survival assay was performed by adding 10 µL of WST-1 (water-soluble tetrazolium salt) from the commercially available EZ-Cytox kit (DoGen, Seoul, Korea) with 90 µL of serum-free medium and incubating the plates for an additional 1 h at 37 °C, according to the manufacturer’s protocol. Absorbance at 450 nm and reference absorbance at 600 nm was measured using a microplate reader (Bio-Tek, Winoosky, VT, USA) and read using Gen5 software (Bio-Tek, Winoosky, VT, USA).

### 2.4. Annexin V/PI Apoptosis Assay

The U251-MG and CRT-MG cells were plated in 6-well plates at a density of 5 × 10^3^ cells/well. Cells were treated with AXT for 3 h prior to treatment with TRAIL and incubated for 16 h in an incubator. After incubation, the cells were gently washed twice with DPBS and trypsinized using trypsin-EDTA (Gibco, Waltham, MA, USA). Subsequently, the cells were stained with fluorescein isothiocyanate (FITC)-conjugated annexin V and propidium iodide (PI) using the commercially available FITC annexin V Apoptosis Detection Kit I (BD Pharmingen, Franklin Lakes, NJ, USA). Cells were divided into viable, early apoptotic, late apoptotic, and necrotic cells based on their staining using the ACEA NovoCyte Flow Cytometer (Acea Bioscience, San Diego, CA, USA) and the sorted NovoExpress software.

### 2.5. JC-1 Mitochondrial Potential Assay

The U251-MG and CRT-MG cells were plated and treated as described for the apoptosis assay. After cell collection by trypsinization, cells were washed in prewarmed DBPS at 37 °C and collected by centrifugation at 1000× *g* for 5 min at room temperature (RT). Cells were resuspended in 500 µL of prewarmed DPBS by gentle tapping. A stock solution of 2 mM JC-1 (Enzo Life Science, NY, USA) dissolved in DMSO was added at a final concentration of 2 µM, and resuspended cells were incubated for 30 min at 37 °C. After incubation, the cells were centrifuged at 1000× *g* for 5 min and resuspended in DBPS. Flow cytometry analysis was performed to detect green (FITC, detection at 530 nm) and red (PerCP, detection at 675 nm) fluorescence.

### 2.6. Cellular ROS Measurement by DCFH-DA Staining Assay

U251-MG and CRT-MG cells were seeded and treated as described for the apoptosis and mitochondrial assays. Collected cells were washed in prewarmed DPBS and incubated with 2′, 7′-dichlorofluorescin diacetate (DCFH-DA; Enzo Life Science, Farmingdale, NY, USA) dissolved in DMSO to a final concentration of 20 µM for 30 min at 37 °C and kept in the dark before measurement by flow cytometry to detect FITC. Cells were treated with 1% H_2_O_2_ (Fisher Scientific, Waltham, MA, USA) as a positive control.

### 2.7. SOD Enzyme Activity Assay

Superoxide dismutase (SOD) enzyme activity was measured using a commercially available OxiTec^TM^ SOD assay kit (Biomax, Seoul, Korea). Briefly, cells were seeded and treated in 6-well plates under the same conditions as described above. Cells were lysed in 50 µL RIPA buffer (MilliporeSigma, Burlington, MA, USA) at RT for 5 min and centrifuged at maximum speed (10,000× *g*) for 30 min at 4 °C. Then, the supernatant was harvested. Total protein concentration of the lysates was determined using a DC protein assay (Bio-Rad, Hercules, CA, USA). SOD activity in the lysates was measured using a microplate reader at an absorbance at 450 nm according to the manufacturer’s protocol. The activity of SOD in the samples was normalized to the total protein concentration of each sample.

### 2.8. RT-qPCR

Cells were harvested, and RNA was extracted using the RNeasy Mini kit (Qiagen, Hilden, Germany). First-strand cDNA was synthesized using AccuPower^®^ RT Premix (Bionner, Daejeon, Korea). The following primers were used with SYBR qPCR Mix (CellSafe, Yongin, Korea) on a CFX96 Real-Time System (Bio-Rad, MA, USA): SOD1 (forward: 5′-cgcacactggtggtccatgaaaaagc-3′, reverse: 5′-acaagccaaacgacttccagcgt-3′); SOD2 (forward: 5′-cactgcaaggaacaacaggcctta-3′, reverse: 5′-tgaaggtagtaagcgtgctcccac-3′); GAPDH (forward: 5′-ccctcaacgaccactttgtc-3′ and reverse: 5′-ccaccaccctgttgctgta-3′). Data were analyzed using the Pfaffl method [30].

### 2.9. Immunoblotting Assay

Cells were seeded, treated, and harvested using a method similar to that described above. Cells were lysed using RIPA buffer, and the supernatant protein concentration was assayed using a DC protein assay. Cell lysates (50 µg) from each treated well were fractionated by SDS-polyacrylamide gel electrophoresis and transferred onto polyvinylidene difluoride (PVDF) membranes. After blocking for 1 h in 5% non-fat milk at RT, membranes were incubated for 24 h with Caspase-3 (D3R6Y) antibody (#14220) purchased from Cell Signaling (Danvers, MA, USA) or actin (C4) antibody (sc-47778) purchased from Santa Cruz (Dallas, TX, USA). After incubation with primary antibodies, membranes were incubated with horseradish peroxidase (HRP)-conjugated secondary antibodies at RT for 2 h, and bands were detected using a C-digit Blot Scanner (Li-cor, Lincoln, NV, USA).

### 2.10. Construction of a SOD2 Overexpression Plasmid and Transient Transfection

SOD2 was amplified from PCR of U87-MG cells first-strand cDNA pooled as described above using the primers hSOD2_HINDIII_F: 5′-CTTAGGCAAGCTTCGatgttgagccg-3′ and hSOD_BAMHI_R: 5′-CGTCCGGGTACCttactttttgcaagc-3′. PCR was purified and digested with BamHI and HindIII purchased from Enzynomics (Daejeon, Korea) and subsequently ligated into pEGFP-C1 (Clonetech, Mountain View, CA, USA), transfected, and selected in *E. coli*. After selection in kanamycin and Sanger sequencing to validate positive clones, plasmids were isolated using the GeneAll Mini Kit (Ecocell, Deajon, Korea). Purified plasmids were transfected into U251-MG cells using Lipofectamine 2000 (Thermo Fischer, Waltham, MA, USA). Cells were maintained in G418 for three passages, and RT-qPCR was performed to verify SOD2 upregulation prior to cell seeding for AXT and TRAIL treatment.

### 2.11. Gepia Data Set Analysis

GEPIA is a web-based portal that enables visualization of patient data from The Cancer Genome Atlas (TCGA) and Genotype-Tissue Expression (GTEx) projects [31]. The screening condition for this study was “Gene: SOD2”, “Dataset: GBM” for expression and survival. Survival plots were computed based on disease-free survival (RFS) for patients in two SOD2 expression groups cutoff as quartile (high and low) with hazard ratio (HR) calculated based on the Cox PH model.

### 2.12. Statistical Analysis

All experiments were performed in three biological replicates with more than three technical replicates. Results are expressed as the mean ± SD. Results with statistical significance were assayed by Student’s t-test with two tails compared to the control.

## 3. Results

### 3.1. AXT Treatment of Selected GBM Cell Lines Do Not Trigger Cell Death

**Figure 1:**
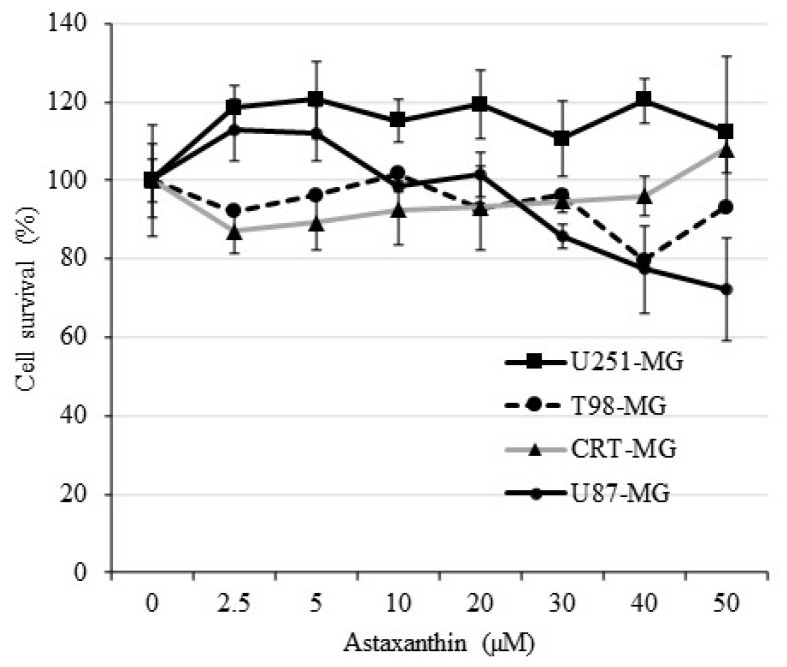
AXT treatment to GBM cells does not affect cell viability. The U251-MG, T98-MG, U87-MG, and CRT-MG were seeded in 96-well plates and treated for 24 h with AXT at concentration from 0 to 50 µM in serum-free media. Cell survivals are expressed in %, compared to 0.1% DMSO-treated control as 100%. Results are shown as average with SD.

AXT was proposed to have anti-cancer properties by upregulating proapoptotic proteins (Bax/Bad and PARP) and downregulating anti-apoptotic proteins (Bcl2, p-Bad, and survivin) in colorectal cancer, melanoma, and gastric carcinoma cell lines [21]. Therefore, to assay the effect of AXT in GBM cell lines, we treated with AXT for 24 h at 2.5 to 50 µM concentration for four GBM cell lines U251-MG, T98G-MG, CRT-MG, and U87-MG. Except for U87-MG, which showed a minor apoptotic effect (around 80% survival) at concentrations above 20 µM, AXT had no proapoptotic effect in the GBM cell lines U251-MG, T98G-MG, and CRT-MG (Figure 1).

These results show that unlike previously investigated cancer cell lines [21], the anti-cancer ability of AXT is restricted in GBM cell lines. This is consistent with our previous study showing that AXT has only a moderate apoptotic effect and shows beneficial effects in GBM cells [32].

### 3.2. AXT Pretreatment Sensitized U251-MG and T98-MG to TRAIL Treatment

**Figure 2:**
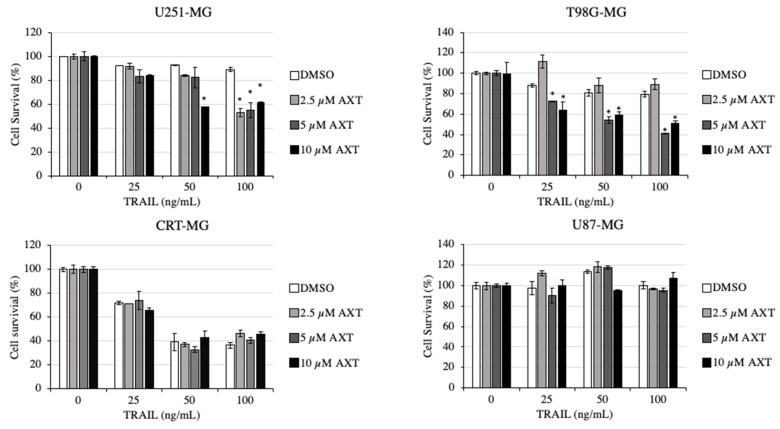
Astaxanthin specifically sensitizes U-251-MG and T98G-MG cell lines to TRAIL treatment while not effective to CRT-MG and U87-MG. GBM cell lines were pretreated with AXT from 2.5 to 10 µM or 0.1% DMSO as control for 3 h prior to treatment with TRAIL from 0 to 100 ng/mL for 16 h. Cell survivals are expressed in % as compared to 0.1% DMSO-treated control as 100%. Experiments were performed in three biological replicates, and representative results are shown with average and SD. * *p* < 0.05, significantly different from the DMSO group (Dunnett’s test).

U251-MG and T98G-MG are TRAIL-sensitive compared to U87-MG [33,34], while to our knowledge, CRT-MG response to TRAIL was not evaluated. To determine whether AXT properties affect the response of GBM cell lines to TRAIL, we pretreated GBM cell lines with 2.5 to 10 µM AXT for 3 h prior to TRAIL treatment for 24 h (Figure 2).

In U251-MG and T98G-MG, AXT treatment above 2.5 and 5 µM, respectively, was effective in increasing the proapoptotic effect of TRAIL. On the other hand, in both CRT-MG and U87-MG, AXT pretreatment had no or minor effect in sensitizing cells to TRAIL treatment. This result suggests that AXT affects the apoptotic pathway induced by TRAIL in specific GBM cell lines.

### 3.3. Apoptotic Pathway Response to AXT and TRAIL Diverge between U251-MG and CRT-MG

**Figure 3:**
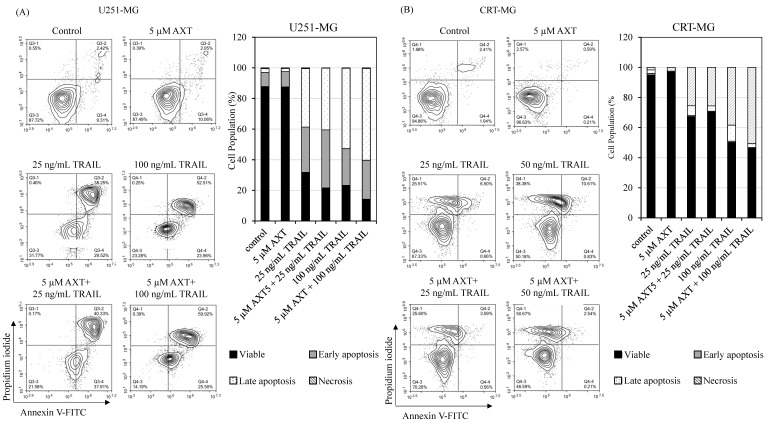
Pretreatment of AXT sensitizes U251-MG cells to TRAIL treatment by increasing apoptotic cells population. Sensitization was not observed in CRT-MG. (**A**) U251-MG cells and (**B**) CRT-MG cells were plated in 6-well plates and treated with 5 µM AXT or 0.1% DMSO as pretreatment for 3 h prior to TRAIL 0, 25, or 50 ng/mL for 16 h. A total of 10,000 cells were analyzed per sample, and cell populations were divided based on staining as Annexin V^−^/PI^−^ (Viable), Annexin V^+^/PI^−^ (Early apoptosis), Annexin V^+^/PI^+^ (Late apoptosis), and Annexin V^−^/PI^+^ (Necrosis). Percentages of population are shown as graphs on the right of each cell line.

To compare the effect of AXT on the apoptotic pathway induced by TRAIL, we performed annexin V/PI staining assay in AXT pretreated and TRAIL-treated U251-MG and CRT-MG cells (Figure 3).

In U251-MG cells, AXT treatment did not affect the apoptotic stage, and TRAIL increased cell population in both the early and late apoptotic stages, as expected. However, upon TRAIL treatment, the population at the early apoptotic stage increased in cells pretreated with AXT (Figure 3A). This suggests that, while having no apoptotic effect by itself, AXT increased the TRAIL proapoptotic effect in U251-MG. On the other hand, in CRT-MG, AXT treatment ameliorated cell survival to a minor extent, as the viable population slightly increased in control cells. Upon TRAIL treatment, the apoptotic cell population increased, as expected. However, unlike U251-MG, TRAIL pretreatment with AXT prior to TRAIL treatment had no or minor effect on the total viable cell population, albeit more apoptotic cells were observed to be in the late apoptotic stage than the early apoptotic stage compared to TRAIL-only-treated cells (Figure 3B). All in all, compared to TRAIL-treated cells, AXT+TRAIL-treated cells consistently show a decrease in viable population in U251-MG (Figure 3A) but not in CRT-MG (Figure 3B).

This result is consistent with the survival assay results and suggests that AXT pretreatment sensitized U251-MG to TRAIL treatment, with only a minor effect on CRT-MG cell viability.

### 3.4. Mitochondria Potential Is Differentially Affected by AXT Treatment between U251-MG and CRT-MG Cells

**Figure 4:**
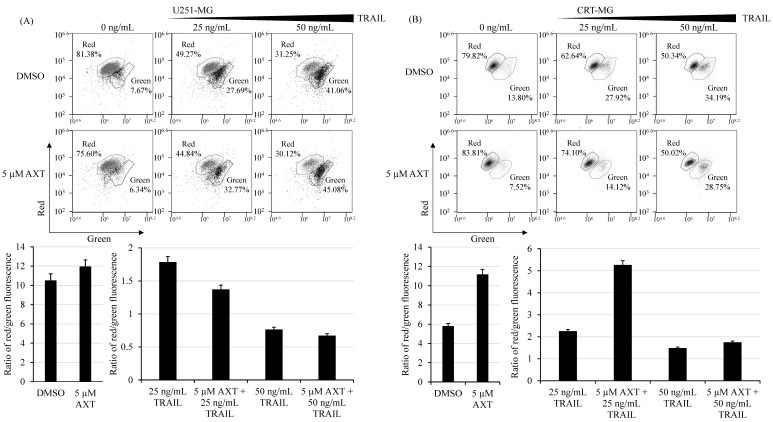
AXT treatment increases while TRAIL treatment decreases mitochondrial inner membrane potential in U251-MG and CRT-MG, while pretreatment of AXT prior to TRAIL treatment further decreased mitochondrial inner membrane potential specifically only in U251-MG. (**A**) U251-MG and (**B**) CRT-MG were plated and treated with 5 µM AXT or 0.1% DMSO as control for 3 h prior to TRAIL treatment of 25 or 50 ng/mL for 16 h. Mitochondrial inner membrane potential was assayed by staining cells with JC-1, and red/green fluorescence was measured by flow cytometry. To better visualization, histograms were divided in two for both experiments as range of control (DMSO) and TRAIL-treated samples differ greatly. Three independent experiments were performed, and 10,000 cells were counted in each experiment. A representative figure for both cell lines is shown with means and SDs presented above.

It was previously established that cross-talk between TRAIL signaling and mitochondrial-mediated apoptosis exists [35] and that upon TRAIL treatment, mitochondrial potential decreases in cancer cells, correlating with selective TRAIL-induced apoptosis in cancer cells [36]. We investigated the effect of AXT treatment on membrane potential by performing a JC-1 assay in U251-MG (Figure 4A) and CRT-MG (Figure 4B).

The red/green fluorescence ratio is an indicator of healthy mitochondria in this assay. As it is shown in Figure 4, AXT increased mitochondrial potential, although it was more effective in CRT-MG. Interestingly, 5 µM AXT was sufficient to compensate 25 ng/mL TRAIL-treated CRT-MG mitochondrial inner membrane potential change, while it unexpectedly further decreased TRAIL-treated U251-MG mitochondrial inner membrane potential. This suggests that while AXT by itself increased mitochondrial health in GBM cell lines, its treatment prior to TRAIL resulted in further mitochondrial inner membrane potential decrease in U251-MG cells.

### 3.5. TRAIL-Induced ROS Was Downregulated by AXT Treatment, but at Significantly Higher Extent in CRT-MG Compared to U251-MG

**Figure 5:**
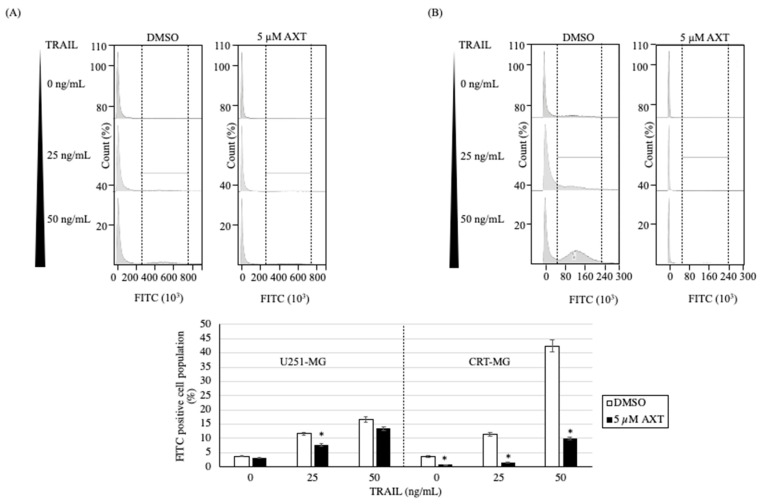
ROS is downregulated by AXT treatment and upregulated by TRAIL treatment, while to a lesser extent in U251-MG compared to CRT-MG. (**A**) U251-MG cells and (**B**) CRT-MG cells were treated with 5 µM AXT or 0.1% DMSO in a 6-well plate as control for 3 h prior to TRAIL treatment of 25 or 50 ng/mL for 16 h. Intracellular ROS was measured by staining cells with DCFD-DA and measuring green fluorescence by flow cytometry. Experiments were performed in three independent replicas, and 20,000 cells for each treatment were measured, and a representative figure is shown. Green fluorescence intensity area was defined using H_2_O_2_ as positive control (data not shown), and ROS-positive population percentage as means with SDs were presented as graphs below each cell line representative figure. Significance was assessed by Student’s *t*-test. * *p* < 0.05.

To investigate cellular ROS changes upon AXT and TRAIL treatment, we performed a DCFH-DA assay in U251-MG (Figure 5A) and CRT-MG (Figure 5B). While U251-MG and CRT-MG baseline ROS were similar (3.73% and 3.26% FITC-positive population, respectively) in untreated cells, U251-MG cells were less affected by AXT pretreatment compared to CRT-MG (decrease in ROS to 3.26% compared to 0.76%, respectively). As TRAIL induced ROS production in both cell lines, the effect of AXT was more apparent in CRT-MG cells. This shows that in high SOD2 expressing CRT-MG cells, AXT treatment is effective in reducing ROS, while in low SOD2 expressing U251-MG, AXT treatment is less effective. ROS decreased around 1.1× to 1.5× in U251-MG, while it decreased around 4.5× to 5.0× in CRT-MG.

### 3.6. Differential Expression of SOD2 in GBM Cell Lines

**Figure 6:**
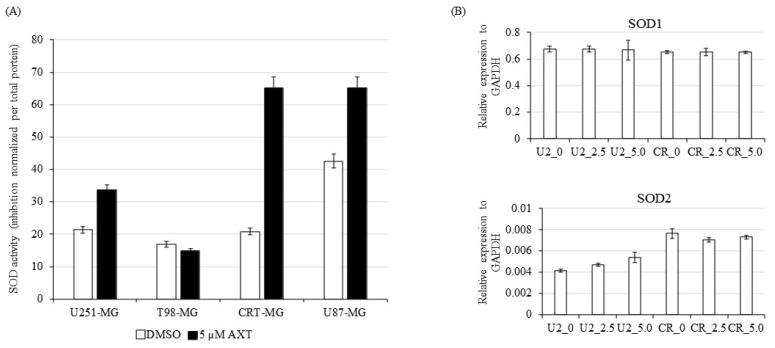
GBM cell lines have differential SOD activity, partially due to differential SOD2 expression. (**A**) U251-MG, T98G-MG, CRT-MG, and U87-MG cells were grown in a 6-well plate and treated with 0.1% DMSO or 5 µM AXT for 24 h in serum-free media before harvesting. After lysis, SOD activity was assayed by kit. Activity of lysates was normalized by total protein content of each treatment. Experiments were performed in three independent replicates, and means with error bars for standard deviation are presented. (**B**) SOD1/2 expression in U251-MG and CRT-MG treated for 0.1% DMSO (0), 2.5 µM AXT, or 5.0 µM AXT for 24 h, which were then harvested, RNA extracted, and cDNA synthesized. RT-qPCR was performed, and expression was assayed using the Pfaffl method with GAPDH expression as control. Three independent replicates were performed, and a representative figure is shown for means and standard error bars for three repeats. U2_0: U251-MG treated with 0.1% DMSO, U2_2.5: U251-MG treated with 2.5 µM AXT, U2_5.0: U251-MG treated with 5.0 µM AXT, CR_0: CRT-MG treated with 0.1% DMSO, CR_2.5: CRT-MG treated with 2.5 µM AXT, and CR_5.0: CRT-MG treated with 5.0 µM AXT.

AXT is known for its ROS-scavenging properties [15], and ROS is known to be the central driver of mitochondrial dysfunction, resulting in a decrease in mitochondrial potential [37]. SODs are highly conserved antioxidative enzymes that catalyze the dismutation of ROS to H_2_O_2_ in eukaryotic cells [38]. As GBM cell responses to AXT pretreatment were different upon TRAIL treatment, we investigated the activity of SOD in the tested GBM cell lines using an SOD assay kit (Figure 6A). SOD activity in GBM cell lines was low overall for U251-MG, T98G-MG, and CRT-MG. However, upon AXT treatment, SOD enzymatic activity increased greatly for CRT-MG and U87-MG, while U251-MG and T98G-MG SOD activity remained relatively low.

Among the three known SODs in the eukaryotic system, we investigated the expression of SOD1 and SOD2, as both were reported to be involved in the regulation of the mitochondrial apoptosis pathway [39]. To this end, we performed RT-qPCR in the AXT pretreatment-sensitive cell line U251-MG and the AXT pretreatment unsensitive cell line CRT-MG. SOD1 RNA expression levels were similar in U251-MG and CRT-MG and were not controlled by AXT, as no statistical difference was observed. On the other hand, SOD2 RNA expression was consistently lower in U251-MG than in CRT-MG and was slightly affected by AXT, while not in a statistically significant manner (Figure 6B).

U251-MG has a low SOD activity and a low SOD2 RNA expression, therefore suggesting a putative relationship between low SOD2 enzyme activity and AXT triggered TRAIL sensitivity.

### 3.7. Increasing SOD2 Expression Lowered U251-MG Cell Sensitization to TRAIL by AXT Treatment

**Figure 7:**
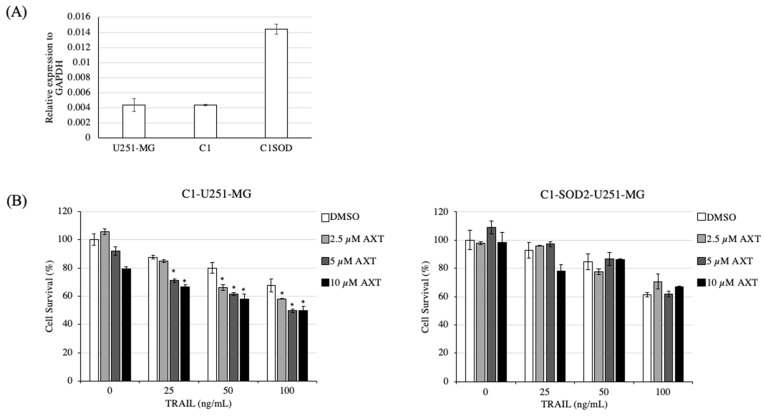
Transient SOD2 overexpression in U251-MG abolished AXT sensitization to TRAIL treatment. (**A**) U251-MG cells were transfected with C1-SOD2-GFP plasmid or C1-GFP plasmid, and SOD2 expression was analyzed by RT-qPCR to monitor transfection efficiency. (**B**) C1-U251-MG (control) and C1-SOD2-U251-MG were plated for treatment with AXT and TRAIL at conditions as described above. Experiments were performed in three independent replicas, and representative figures are presented for both cell lines. Cells survivals were presented as means and SD with DMSO-treated cells as control (100% survival). * *p* < 0.05, significantly different from the DMSO group (Dunnett’s test).

To further investigate whether SOD2 is related to sensitization by AXT to TRAIL treatment, we overexpressed SOD2 in U251-MG cells using a C1-GFP vector (Figure 7). As expected by our hypothesis that AXT and low SOD2 activity result in increased TRAIL sensitivity, upregulating SOD2 expression in U251-MG should decrease AXT sensitization. Indeed, compared to the control transformed with empty C1-GFP vector, U251-MG expressing C1-SOD2 lost their sensitization by AXT upon TRAIL treatment (Figure 7).

This suggests that the sensitization of U251-MG AXT cells to TRAIL treatment was due to low SOD2 expression.

## 4. Discussion

Because of its anti or pro-oxidative properties, it can be inferred that the role of AXT in cancer cells is closely related to ROS and mitochondrial potential. Recent studies have shown that in cancer cells, AXT can regulate ROS production, and its pro-oxidative features are linked to proapoptotic effects in cancer cells selectively while having antioxidative features linked to protective effects in nearby healthy cells [15]. However, as the pathways involved in AXT treatment are not yet well understood, the outcome of AXT treatment in cancer is difficult to predict. For example, in breast cancer, it was shown that co-treatment of AXT with the anti-cancer drug carbendazim decreased ROS production in treated breast cancer cells, possibly hindering the ROS-inducing anti-cancer drug effect [40]. In GBM cells, it was previously shown that AXT up to 150 µM does not affect cell viability but affects cell migration by downregulating matrix metalloproteinase (MMP)-2 in 50 µM AXT-treated A172 cell line [41]. We recently showed that in U251-MG cells, AXT cell toxicity was not apparent when treated for 24 h and rather had a hormesis (proliferative) effect at concentrations below 10 µM by affecting cyclin-dependent kinase and down-regulation of p53 [32]. However, another study showed that AXT moderately affected cell viability (down to around 80%), cell migration, and upregulated ROS production in U251-MG at concentrations over 10 µM [42]. This discrepancy is probably because apoptosis in the above-mentioned study was performed in U251-MG cells treated for 92 h, while we assayed AXT activity for 24 h for this study, as our previous study shows that treatment for 72 h has apoptotic effect in this cancer cell line [42]. Overall, while long-term AXT treatment might have pro-oxidative and apoptotic effects on GBM cells, short and low AXT did not affect GBM cell viability to a significant extent. Notably, while affecting cell viability, AXT IC_50_ was not reached at concentrations over 100 µM in gastric cancer cell lines [43], suggesting that while AXT indeed acts as a proapoptotic agent in cancer, as has been suggested by several in vivo experiments in mice [44,45,46], their outcome might be moderate when investigated in cancer cell lines as treatments are limited in concentration and time. As mentioned in the introduction, according to FEEDAP, the ADI of AXT is 0.2 mg/kg for human consumption. Studies in human intake of AXT show that AXT intake for a single dose up to 100 mg, that is presumably close to the ADI, increased AXT concentration in plasma up to 2.2 µM [47] or intake for 8 weeks up to 8 mg of AXT increased AXT concentration in plasma up to 0.13 µM [48]. Interestingly, AXT intake was shown to have an effect on brain tissue in several organisms, including humans [49], and in rats, oral feeding of 50 mg/kg for 10 days resulted in the detection of a total of 33.87 ± 6.93 ng per g of brain tissue (equivalent to 0.056 µM) therefore demonstrating that indeed AXT can cross the blood-brain barrier and can have a biological effect in brain tissue via oral injection. While experiments with cell lines and concentration of plasma do not exactly correlate with the effective concentration for treatments [50], it can be inferred from the above-mentioned studies that as AXT concentration increased in plasma to a maximum of 2.2 µM upon oral injection, and as that effective GBM cell apoptosis was induced at a concentration higher than 8 µM for 72 h [32] or 10 µM for 92 h [42], that in conclusion, to induce AXT anti-cancer effect and avoid putative hormesis effect [42], drug delivery should be considered to be induced in alternative methods beside oral injection. On the other hand, as TRAIL sensitization in this study was performed at relatively lower and shorter exposure that did not trigger cell apoptosis, there is a higher possibility that oral absorption of AXT might have a positive effect that sensitizes GBM tissues to TRAIL treatment. All in all, while several studies, including this study that shows AXT potential in TRAIL sensitization, suggest that AXT might be a useful tool in treating cancer, more careful investigation and method of delivery should be studied as treatment below effective concentration might have no effect or even negative consequences such as hormesis effect in cancer.

It is well established that cross-talk between TRAIL-induced extrinsic apoptosis and mitochondria-associated intrinsic apoptosis exists, as TRAIL/TRAIL-R-activated caspase-8 can trigger mitochondrial apoptotic signals. This results in cytochrome c release and an increase in ROS that triggers ROS release [51], altogether increasing caspase-3 cleavage, thereby amplifying the apoptotic signal [52,53]. Interestingly, we found that AXT affected selective GBM cell line sensitization upon TRAIL treatment. TRAIL sensitization of cancer cells by plant-produced antioxidants, such as flavonoids, is well known [44], but their mechanisms are still not well understood. In our study, we found that from the four cell lines assayed, U251-MG and T98G-MG both were sensitized to TRAIL treatment, with only moderate effects in CRT-MG and U87-MG (Figure 2). As annexin/PI assays show, an increase in apoptotic cell population by pretreatment of AXT in U251-MG in TRAIL-treated cells, but not AXT alone. This suggests that the cell viability change by AXT enhanced TRAIL-mediated apoptosis (Figure 3A) rather than triggering necroptosis, as was observed in a gastric cancer cell line [20], or individually triggering apoptosis, as was shown in a colon cancer cell line [19].

AXT was previously shown to affect apoptosis controlled via mitochondrial potential in hepatocellular cancer cell lines [54], and in GBM cells, we showed low or opposite effects when treated individually. The percentage of viable cell population in CRT-MG cells treated with AXT was consistently high compared to the control in all annexin V/PI assays performed (Figure 3B, data not shown), although it was not statistically significant as the baseline of untreated cells viable population was high. The beneficial effects of AXT in CRT-MG might be because mitochondrial health increased (Figure 4B) and intracellular ROS decreased (Figure 5B) upon AXT treatment, suggesting that antioxidant properties of AXT have a beneficial effect on CRT-MG, while as our data show that this alleviation was not enough to inhibit TRAIL-induced apoptosis (Figure 2). Nonetheless, together with our previous finding that low-dose AXT triggers cell proliferation and that three of four AXT-treated GBM cell lines’ viability increased above 100% in this study (Figure 1), this suggests that for some GBM cell lines, low and/or short AXT treatments might increase cell survival rather than have apoptotic effects.

While these antioxidant effects of AXT are also observable in U251-MG as ROS decreases and lower mitochondrial potential (Figure 4A and Figure 5A), they appear to be less effective, also shown by the moderate viable cell population increase upon AXT treatment observed by annexin V/PI staining assay (Figure 3A and data not shown). Rather, AXT pretreatment sensitized U251-MG to TRAIL treatment, thereby increasing the number of apoptotic cells (Figure 2 and Figure 3A). In addition, AXT treatment increased caspase-9 cleavage in TRAIL-treated cells (Supplementary Figure S1), indicating that pretreatment with AXT increased TRAIL-induced apoptosis. Interestingly, it was shown that, unlike CRT-MG, AXT pretreatment increased mitochondrial stress in TRAIL-treated U251-MG cells (Figure 4A,B), and it still decreased total cellular ROS in U251-MG, but to a lesser extent than CRT-MG (Figure 5A,B).

Usually, TRAIL treatment and ROS increase correlate with mitochondrial depolarization and apoptosis, as it was previously shown that TRAIL treatment in cancer cells selectively leads to increased mitochondrial ROS and depolarization [36]. Indeed, we also observed that TRAIL treatment resulted in both U251-MG and CRT-MG mitochondrial membrane depolarization (Figure 4) and total ROS cellular increase (Figure 5). However, it was also reported that downregulating ROS in TRAIL-treated pancreatic cancer cells unexpectedly increased early apoptotic cells [25], indicating that the exact relationship between mitochondria and apoptosis is not yet well understood. One possible explanation for the observed ROS decrease while mitochondrial membrane depolarization increases in AXT pretreatment of TRAIL-treated cells is that while total cellular ROS decrease, presumably due to the antioxidant activities of AXT, TRAIL, and AXT together increasing mitochondrial oxidative stress. This is supported by the fact that this sensitivity to AXT was dependent on the mitochondrial matrix-specific antioxidant enzyme SOD2 [39,55,56] expression in U251-MG (Figure 7). However, we cannot rule out the possibility that membrane depolarization increase in AXT/TRAIL-treated U251-MG is ROS-independent. Rather, AXT acts with SOD2 in a pathway involving other mitochondrial apoptotic proteins such as Bcl-2 [57] or BAX [58], as previously shown. Additional experiments are needed to determine the pathways involved in sensitizing TRAIL-triggered apoptosis in GBM cell lines with low mitochondrial antioxidative capacity.

Overall, our results suggest that while cross-talk between TRAIL signaling and ROS increase exists, an independent ROS/mitochondria potential-mediated apoptosis pathway involving SOD2 might be partially controlled by AXT (see graphical abstract).

## 5. Conclusions

We showed that the antioxidant enzyme SOD2 is involved in TRAIL sensitization via a pathway involving mitochondrial potential change leading to amplification of the TRAIL apoptosis signal in GBM cell lines. This suggests that AXT can affect the TRAIL-induced pathway, not by its antioxidative ability in the cytosol but rather by a specific pathway involving the mitochondrial organelle. As we only assayed caspase 9 activity in U251-MG, more mitochondrial-associated proteins activation or release related to the apoptotic pathway such as BCL2, BAX, or cytochrome c should be assayed to investigate the mechanism of apoptosis sensitization by AXT related to SOD2.

Therefore, while our results suggest that adding AXT might be effective in GBM treatment, it might be only in specific conditions, that is, low SOD2 activity. While additional experiments such as downregulating SOD2 should be performed, these findings suggest that the efficiency of AXT as a treatment to enhance anti-cancer drug results might be affected by SOD2 expression in patients. Therefore, AXT usage and effects should be carefully monitored, especially in GBM patients, since overall SOD2 expression in GBM tumors of patients is higher compared to nearby healthy tissue (Supplementary Figure S2A), and overall disease-free survival was lower in the high SOD2 expressing patient group with a hazard ratio of SOD2 close to 1.8 (Supplementary Figure S2B).

## Data Availability

Data are contained within the article and Supplementary Materials.

## Supplementary Materials

The following supporting information can be downloaded at: https://www.mdpi.com/article/10.3390/antiox11020375/s1, Figure S1. Western blot analysis of Caspase-3 protein in AXT and TRAIL-treated U251-MG cells, Figure S2. GBM patients’ tumor (T) and normal (T) brain tissues SOD2 expression (A) and disease-free survival plot with hazard ratio (HR) (B) from GEP.
