# Supplementary material for: Astaxanthin Sensitizes Low SOD2-Expressing GBM Cell Lines to TRAIL Treatment via Pathway Involving Mitochondrial Membrane Depolarization
Source: Antioxidants (Basel). 2022 Feb 13;11(2):375. doi: 10.3390/antiox11020375 (PMC8869337; doi:10.3390/antiox11020375)
Supplement: Supplementary file 1 [file antioxidants-11-00375-s001.zip › antioxidants-1531499-supplementary.pdf]

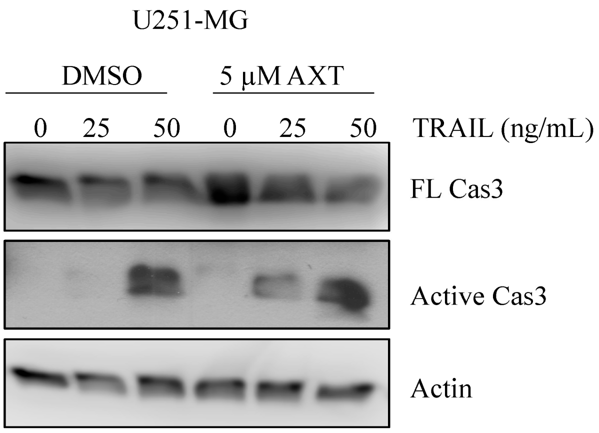

Figure S1. Western blot analysis of Caspase-3 protein in AXT and TRAIL-treated U251-MG cells.

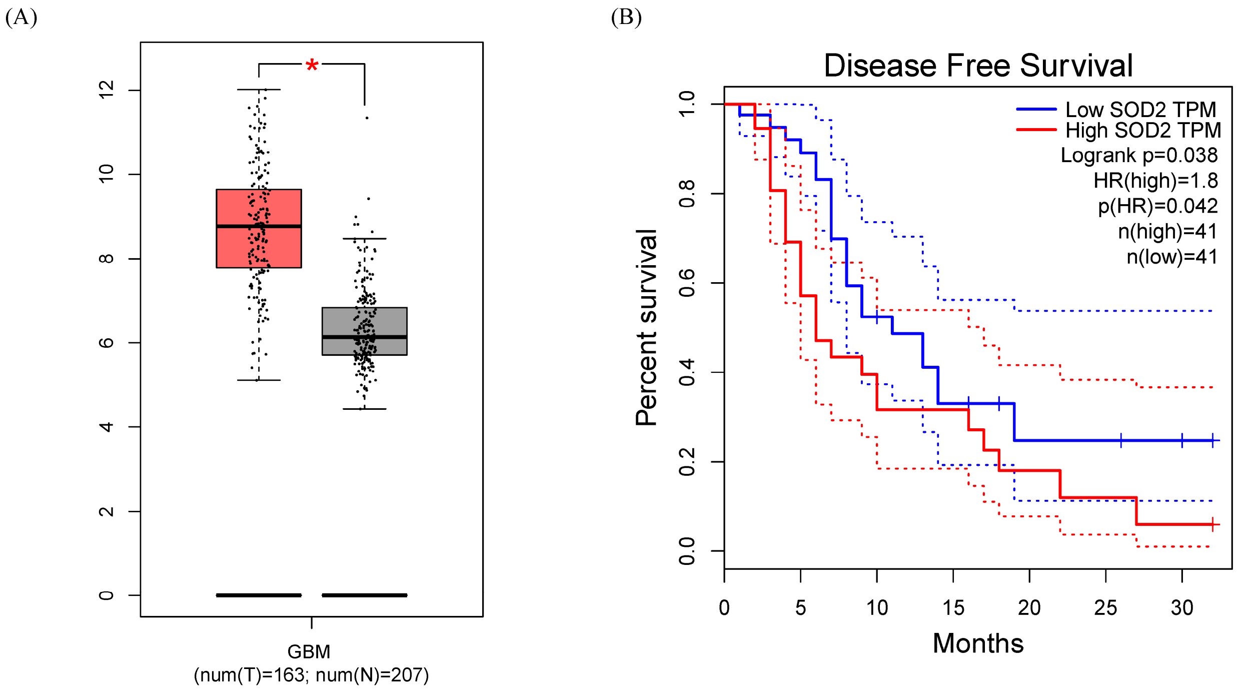

Figure S2. GBM patients' tumor (T) and normal (T) brain tissues SOD2 expression (**A**) and disease-free survival plot with hazard ratio (HR) (**B**) from GEP.
